# Episodic zircon age spectra mimic fluctuations in subduction

**DOI:** 10.1038/s41598-018-35040-z

**Published:** 2018-11-30

**Authors:** Mathew Domeier, Valentina Magni, Mark W. Hounslow, Trond H. Torsvik

**Affiliations:** 1 Centre for Earth Evolution and Dynamics (CEED), University of Oslo, Oslo, Norway; 2 Lancaster Environment Centre, Lancaster University, Lancaster, United Kingdom; 3 Geodynamics Team, Geological Survey of Norway, Trondheim, Norway; 4 School of Geosciences, University of Witwatersrand, Johannesburg, South Africa

## Abstract

Decades of geochronological work have shown the temporal distribution of zircon ages to be episodic on billion-year timescales and seemingly coincident with the lifecycle of supercontinents, but the physical processes behind this episodicity remain contentious. The dominant, end-member models of fluctuating magmatic productivity versus selective preservation of zircon during times of continental assembly have important and very different implications for long-term, global-scale phenomena, including the history of crustal growth, the initiation and evolution of plate tectonics, and the tempo of mantle outgassing over billions of years. Consideration of this episodicity has largely focused on the Precambrian, but here we analyze a large collection of Phanerozoic zircon ages in the context of global, full-plate tectonic models that extend back to the mid-Paleozoic. We scrutinize two long-lived and relatively simple active margins, and show that along both, a relationship between the regional subduction flux and zircon age distribution is evident. In both cases, zircon age peaks correspond to intervals of high subduction flux with a ~10–30 Ma time lag (zircons trailing subduction), illuminating a possibly intrinsic delay in the subduction-related magmatic system. We also show that subduction fluxes provide a stronger correlation to zircon age distributions than subduction lengths do, implying that convergence rates play a significant role in regulating the volume of melting in subduction-related magmatic systems, and thus crustal growth.

## Introduction

**Figure 1:**
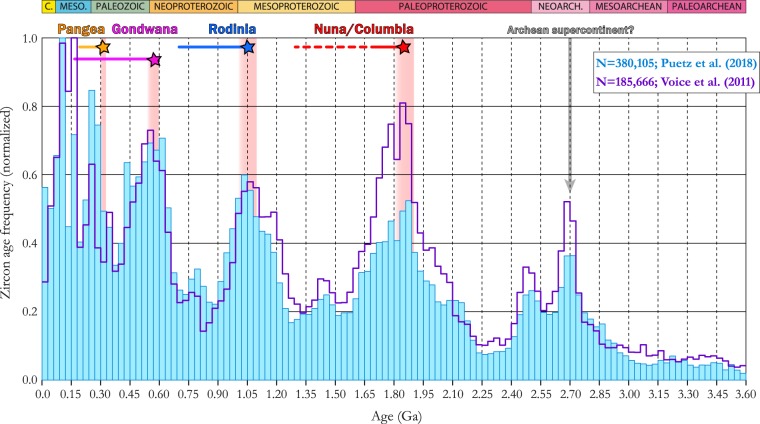
Episodic age distribution of zircons since 3.6 Ga. Blue (purple) histogram shows the age distribution of zircons from the database of Puetz *et al*.^3^ (Voice *et al*.^2^) in 30 Ma bins. Pink bars mark the estimated age of major supercontinent assembly events^44,45^, and the duration of those continents is shown by the colored horizontal bars along the top of the panel.

Physiochemically resilient enough to survive in Earth’s near-surface for billions of years and maintaining a radiogenic isotopic system that can be exploited as a precise chronometer across such timescales, the mineral zircon represents a time-capsule through which aspects of Earth’s extensive history can be glimpsed. In being relatively common in felsic igneous rocks that principally comprise the continents, zircon geochronology is an ideal tool for studying crustal evolution and much work has therefore been invested in trying to understand the temporal distribution of zircon U-Pb ages. That this distribution seems to be episodic was already recognized before the advent of plate tectonics^1^, and while the details of the age ‘peaks’ have been revised in the decades since, the episodic nature of the observed distribution has persisted, and it remains striking in large, modern compilations^2,3^ (Fig. 1). With the prospect of an overwhelming sampling bias vanishing, this episodicity has come to be interpreted in two alternative ways: as a signal of true fluctuations in zircon production^4–6^, or rather an artefact of selective preservation^1,7–9^.

The vast majority of zircons are produced in convergent margin magmatic arcs, so the fluctuating production argument entails that subduction-related magmatism itself has fluctuated through time. Some have interpreted this to imply that subduction (and thus plate tectonics) has operated with variable vigor, possibly driven by changing mantle convection regimes that have been attributed to ‘superplumes’^6^, catastrophic slab avalanching through the transition zone^4^, alternations between single- and two-layer mantle convection^10^, and episodic changes to global plate-mantle coupling^5^. However, direct comparisons between past plate convergence and arc magmatic fluxes have yielded mixed results, for example in Mesozoic to early Cenozoic case studies from the North American Cordillera^11,12^. This has led others to present models that explain arc magmatic fluctuations by periodic processes controlled by shortening in the continental lithosphere^13,14^. A third perspective contends that arc magmatic fluctuations reflected by zircon age distributions are most closely associated with the changing global length of subduction zones^15,16^, and thus that locally varying convergence rates are relatively unimportant in dictating arc magmatic budgets.

In contrast, the selective preservation model argues that the observed zircon age distribution has been strongly biased by dissimilar preservation potentials associated with different tectonic environments, such that the original (unknown) age distribution may have been more uniform^7^. Although active margins are the main factories of continental crust (and zircons), they are also responsible for expunging the crustal record through subduction (both via sediment subduction and subduction erosion; hereafter we refer to these together as ‘tectonic erosion’), and so the contribution of active margin processes to crustal growth through time could be balanced or even negative^17,18^. However, following continental collision, products of the formerly active margin(s) will be shielded from tectonic erosion by the continental mass newly enclosing them, thus safeguarding their preservation^7,19^. A logical deduction is then that zircon preservation should be elevated during times of nascent supercontinent formation, and that zircon age ‘peaks’ could represent a kind-of ‘erosional massif’ rather than a surge in production.

**Figure 2:**
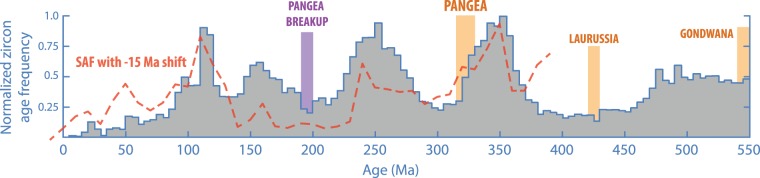
Episodic age distribution of zircons in the Phanerozoic. Age frequency of Phanerozoic detrital zircons from arc environments, from the database of Voice *et al*.^2^. Vertical yellow bars mark major continental assembly events. The red-dashed line shows the subduction area flux of the last 410 Ma, shifted by −15 Ma according to the best-fit correlation lag-time determined by Hounslow *et al*.^20^.

Although these contrasting models of fluctuating production vs. selective preservation are not mutually exclusive, the question of which process has been dominant in shaping the observed zircon age distribution is of great importance to our understanding of the nature and history of plate tectonics, arc magmatism and crustal growth. The production model sees these systems as inherently episodic, whereas the preservation model implies they were likely much more uniform. Thus far, debate on the relative importance of these end-member models has mostly focused on interpretations of the global, Precambrian record, from which the salient coincidence of zircon age peaks and major continental assembly events (Fig. 1) presents a compelling case for the selective preservation model. Yet, for the Phanerozoic, when the history of plate motions and continental collisions is better established, this relationship is not clear, and Hounslow *et al*.^20^ recently showed that the global zircon age distribution appears to mimic fluctuations in subduction (Fig. 2). Here we endeavor to further this production vs. preservation discussion through an exploration of possible links between zircon age distributions and subduction history on a continental scale during the Phanerozoic.

## Phanerozoic Case Studies

To help discriminate between the production vs. preservation end-member models we utilize a continental-scale experimental setup, focused on relatively simple active margins that have avoided major collisional orogenesis. For each selected region, we isolate the Phanerozoic zircon record and subduction history, and compute cross-correlations of these independent time-series to identify any statistically significant linkages.

The western margin of South America and the eastern margin of Australia were selected as case studies because they present relatively simple, long-lived active margins uncomplicated by major continent-continent collisions. The western margin of South America has probably been continuously active since the Early Devonian, excepting a hiatus in the Late Devonian-Early Carboniferous, with only minor terrane collisions occurring along it in the Paleozoic^21^. The eastern margin of Australia was similarly active through most of the Phanerozoic, and although it grew markedly, its growth was due to the opening and closing of backarc basins, the stacking of marginal arcs and the incorporation of small, juvenile island arcs, rather than the accretion of large continental blocks^21–23^.

**Figure 3:**
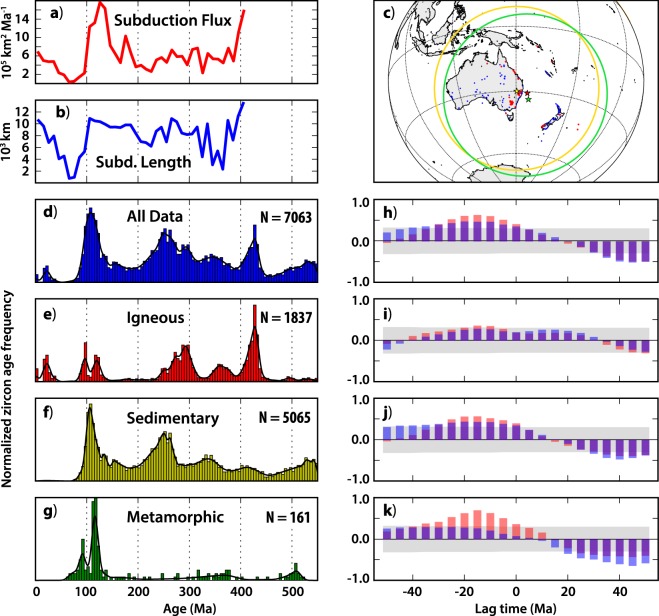
Subduction parameters and zircon age distributions from Oceania. (**a**) Subduction flux and (**b**) subduction lengths calculated in 10 Ma intervals from a full-plate model^24^. (**c**) Zircon localities from the database of Puetz *et al*.^3^; red dots show zircons from igneous hosts, blue dots show zircons from sedimentary and metamorphic hosts. The green star shows the gravitational center of the zircon localities, the red star shows the igneous-only centroid and the yellow star shows the area-based centroid of the zircon localities. The green (yellow) circle shows the 40° search radius about the green (yellow) star. (**d**–**g**) Zircon age distributions from the dataset of Puetz *et al*.^3^, sorted by host rock type. Vertical bars show a normalized histogram in 5 Ma bins; the black line shows a locally adaptive kernel density estimate. ‘N’ is the total number of samples in each set. (**h**–**k**) Cross-correlations of the zircon age distributions (from the panel to the immediate left) and the subduction flux (red bars) and subduction lengths (blue bars) [purple where they overlap], as a function of lag time. For negative lag times, the subduction parameters (flux/lengths) are shifted ahead (in time) of the zircon age distribution. The gray box encloses results that cannot be excluded from the null-hypothesis of no correlation at the p = 0.05 significance level.

**Figure 4:**
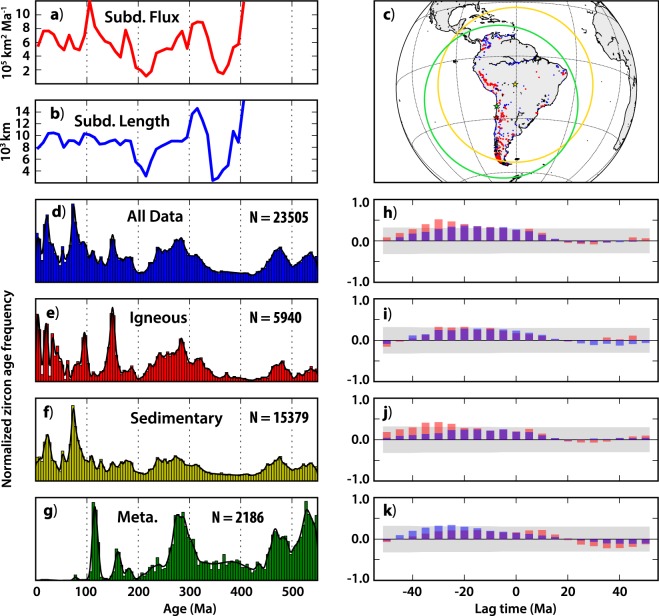
Subduction parameters and zircon age distributions from South America. See Fig. 3 for panel descriptions.

Phanerozoic zircon age records from these two respective regions were extracted from the database of Puetz *et al*.^3^, which, after filtering (see Methods), yielded 7,063 dates from ‘Oceania’ (Australia, New Zealand and proximal Pacific islands) and 23,505 dates from South America. The zircon ages were sorted according to their host rock type as classified by Puetz *et al*.^3^, allowing consideration of ‘igneous’, ‘sedimentary’ and ‘metamorphic’ zircons separately (Figs 3 and 4). The zircon age distributions from both continents are episodic, and although there are obvious similarities and synchronous peaks among the various sub-set distributions from each continent, there are also clear differences. This underscores the importance of considering them independently.

To consider if any links can be drawn between these zircon age distributions and the history of subduction, we calculate the time-dependent length of subduction zones and the subduction flux along eastern Australia and western South America for the last 410 Ma, using a full-plate model^21,24^ (see Methods). Because plate boundaries are continuously networked, the choice of which subduction zones to include in the estimation of subduction length/flux for a given continent is not obvious. The strategy we have adopted is to look within a specific search radius centered on a point that reconstructs with the continent of interest (Fig. S1), and we vary both the location of the point and the size of the search radius to consider how these choices may affect the outcome of the experiment.

Our initial point for each continent coincides with the approximate center of gravity of the Phanerozoic zircon distribution from that continent (Figs 3 and 4). In South America, this initial point lies in the central Andes, ~1,800 km from the geographic center of the continent, whereas in Oceania it is ~2,000 km southeast of the geographic center of Australia. We then select a variety of other starting points centered about the area-based centroid of the zircon distributions, which are closer to the geographic center of each continent. Our initial search radius is 40°, so chosen because (using the initial centroids) it encompasses the bulk of the zircon occurrences and the surface area of the respective continent of interest, while not extending much further beyond (Figs 3 and 4). However, we explore the effect of varying the size of the search radius between 20° and 60°. For each of these various setups, subduction zone lengths and the subduction area flux are calculated at 10 Ma intervals from all subduction segments that enter the search window as both the subduction zones and the search windows are reconstructed through time from 410 to 0 Ma (Fig. S1).

Cross-correlations are then calculated between the zircon age distributions and subduction histories. Because any variation in zircon production that could stem from changes to subduction would expectedly be delayed—according to the (unknown) time required for the magmatic system to respond to the altered subduction conditions, plus melt transit and emplacement times—we conduct these cross-correlations at a variety of lag-times (see Methods). In the following, negative lag-times indicate that changes to subduction occur before a response is observed in the age frequency of zircons. The results of our initial experiments in both Oceania and South America reveal positive correlations between both subduction parameters (length and flux) and all corresponding zircon age distributions, with negative lag times (Figs 3 and 4).

In Oceania, comparison of the subduction parameters with ‘all zircons’ shows a maximum positive correlation at a negative time lag between 10 and 20 Ma. The subduction flux shows a stronger correlation to the zircon age distribution than the subduction lengths do, although the null-hypothesis (of no correlation) can be rejected (*p* < 0.05) for both comparisons at lag times between 0 and −30 Ma (Fig. 3h). Similar results are seen in the comparisons of the subduction parameters with each of the zircon sub-sets, and the subduction flux yields the strongest positive correlation in all cases. The relocation of the search center to other points in the central area of Oceania does not alter these results, nor does changing the radius of the search window (between 20° and 60°), as the shape of the correlation profile remains stable (Figs S2 and S3).

For South America, the null-hypothesis of no correlation between the subduction history and the zircon age distribution can be rejected (*p* < 0.05) for comparisons at lag times between −5 and −35 Ma (Fig. 4h). The strongest correlation between the subduction flux and zircon age frequency occurs at a lag time of −30 Ma. Similarly, in the comparison of the subduction parameters with the igneous and sedimentary zircon sub-sets, the strongest correlation occurs with the subduction flux at lags between −20 and −30 Ma. By contrast, the metamorphic zircon age distribution shows a stronger correlation with subduction length rather than subduction flux, but only correlations at a time lag of −25 and −30 Ma are significant at the 95% confidence level. Experiments with alternative search center locations reveal that this parameter has a greater bearing on the results than was observed in Oceania (see Fig. S4). This is because the cross-correlation profile is mildly bimodal, and the position (in lag-time space) of the largest mode switches from −30 Ma for southern search centers to −15 Ma for northern search centers. However, the general form of the correlation profile is otherwise consistent among these experiments. Experiments with changing the size of the search radius again show more variability than observed in the case of Oceania, but as before the form of the correlation profile remains stable (see Fig. S5). These results indicate that the general outcomes presented are not strongly dependent on the specific search center or search radius selected.

**Figure 5:**
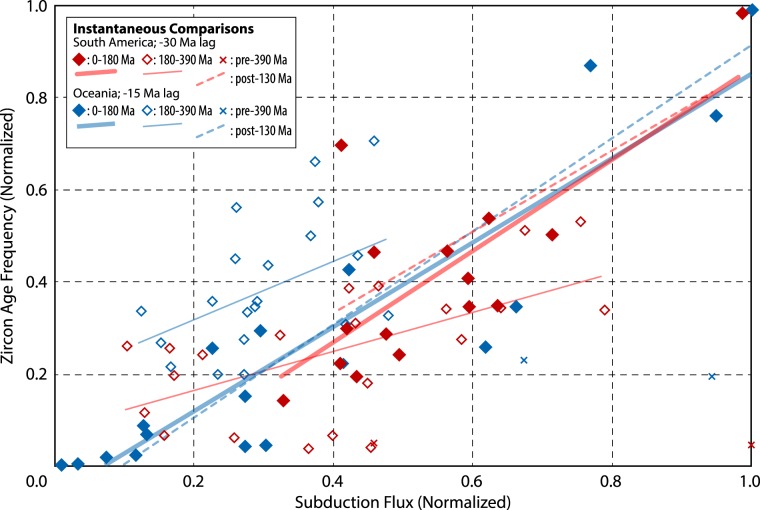
Instantaneous comparisons of subduction flux vs. zircon age frequency data from South America and Oceania. Each symbol represents a time-specific comparison between a subduction flux value and the corresponding zircon age frequency, according to the best-fit time lags from Figs 3 and 4 (i.e. a −30 Ma lag for South America comparisons, and a −15 Ma lag for Oceania comparisons). The zircon age frequency data and subduction flux data are normalized for each continent to allow comparison against one another. Filled (open) diamonds show instantaneous comparisons of data younger (older) than 180 Ma (relative to subduction flux time); crosses show data older than 390 Ma. Separate linear regressions (using ordinary least squares) are shown for data younger than 130 Ma, data between 0–180 Ma, and data between 180–390 Ma. The parameters of correlation for each linear model are reported in Table S1.

Because these experiments were conducted using full-plate kinematic reconstructions that extend beyond the age of the oldest *in situ* oceanic lithosphere in the Pacific (Early Jurassic)^25^—before which time the reconstructed oceanic plates are necessarily entirely synthetic^26^—it is important to consider the temporal changes in these correlations as a function of model time. Figure 5 shows instantaneous comparisons of the subduction flux and zircon age frequency for both South America and Oceania (using the best-fit lag times from Figs 3 and 4), separated into pre- and post-180 Ma (Early Jurassic) time. Linear regressions through all temporal groups of data show positive correlations, but the strongest positive association for each continent is observed in the younger (post-180 Ma) comparisons. The correlations among the pre-180 Ma comparisons, although weaker and less positive, are generally similar, with only results from the earliest model interval (Early Devonian) presenting clear outliers to the younger trends. An even more conservative analysis, considering only the times for which a global moving hotspot reference frame is available^27^ (0–130 Ma), yields relationships nearly indistinguishable from those of the last 180 Ma (Fig. 5).

## Discussion

Our results suggest that along eastern Australia and western South America—margins unperturbed by major continental collisions since the early Paleozoic—the observed Phanerozoic zircon age distributions are relatable to the regional subduction flux with a lag time of ~10–30 Ma (subduction leading zircon production). Before proceeding with an interpretation of these findings, we underscore a significant caveat associated with them: namely, that there are unquantified uncertainties present in both the plate model and zircon age distributions.

Uncertainties in the plate tectonic model worsen backward in time, particularly prior to the Late Jurassic, before which time there exists no direct plate reference frame for the Pacific-Panthalassic basin, and very little *in situ* oceanic lithosphere. Uncertainties on the paleolatitudinal and azimuthal reconstruction of the continents can be estimated from paleomagnetic data for earlier times, but errors associated with the reconstruction of the (synthetic) oceanic plates are not quantifiable^26^. In younger times (especially after 130 Ma), absolute reference frames and marine records provide increasingly numerous and more robust constraints with decreasing age, but there may still be significant flaws in the reconstruction of the oceanic plates associated with unresolved intra-oceanic subduction histories^28–30^.

With respect to the zircon data, it is unknown how well the sample age distributions mimic the true zircon age distributions of the margins at present-day. Bias may have been introduced by the non-uniform nature of the individual sampling campaigns, by differential erosion, and by the fact that young plutonic bodies have not yet been exhumed, whereas older magmatic units may have already been entirely eroded and either subducted or re-buried in sedimentary basins. Additionally, the relationship of the true zircon age distribution of the margin to magmatic volume fluctuations can be complicated by differential zircon fertility of the arc products through time^31^.

In spite of these notable uncertainties, we consider the emergence of similar correlations from our varied experiments to be indicative of a meaningful link between the subduction flux and zircon age distributions. In the context of the production vs. preservation end-member models, these correlations could either imply that the subduction flux drives fluctuations in arc magmatism or that it controls the preservation potential of zircon along active margins. Our case study margins were so-chosen to minimize the influence of changing zircon preservation potentials that are postulated to respond markedly to subduction termination events associated with continent-continent collision^7^. Thus, an initial conclusion to be drawn is that zircon age ‘peaks’, at least those observed on a continental scale, cannot solely be ascribed to selective preservation linked to continental assembly events.

This does not dispel the importance of selective preservation in our case studies per se, because even along a continuously active margin the preservation potential of zircon could be expected to fluctuate according to changes in the volume of crustal losses to subduction through time. Our experiments are ill-posed to exclude contributions from fluctuating preservation potentials in this (non-collisional) form, and so they may be affecting the computed correlations. To estimate and exclude these contributions would require time-dependent tectonic erosion budgets for each margin spanning hundreds of Ma, which are unfortunately unavailable. Nevertheless, along modern active margins, total crustal material losses to tectonic erosion exhibit no discernable correlation with convergence rates^17,32^. Furthermore, it is not obvious how or why fluctuations in the vigor of subduction would cleanly ‘carve’ pre-existing zircon age distributions in such a way as to mimic the subduction flux with a particular lag time. We therefore contend that the positive correlations observed between the subduction flux and zircon age distributions do not likely arise from selective preservation processes, albeit they may be obfuscated by them.

We prefer the interpretation that the observed correlations are associated with fluctuations in arc magmatism driven by changes in subduction. Because the subduction flux better correlates with the zircon age distributions than do subduction lengths, our results further suggest that the convergence rate, in addition to the length of subduction zones, is an integral variable regulating arc magmatic budgets. But, by what mechanism can the convergence rate modulate arc productivity? Geodynamic models have revealed that the zone of crustal dehydration in the subducting slab may broaden with increasing rates of convergence^33^, allowing the area of melting in the overlying mantle wedge to widen. If this yields a wider magmatic arc at the surface, the total magmatic flux of an arc could be augmented without otherwise requiring an enrichment of the mantle wedge^13^. A second explanation offered by numerical experiments is that higher convergence rates will drive more vigorous convection in the mantle wedge, bringing in hotter, more fertile mantle that may permit greater melting and thus larger magmatic fluxes^34^. Changes in the flow of the mantle wedge can also modify the thermal and viscous structure of both the wedge itself and the basal lithosphere of the overriding plate, and may thereby enhance (or suppress) decompression melting that may contribute to the arc magmatic budget^35,36^.

Interpretation of the observed ~10–30 Ma lag times is challenging. According to present-day estimates, average upper-mantle slab sinking rates mostly range between ~1–10 cm/yr^37^, so the transit of lithosphere from the trench to the dehydration-melt generation zone at ~100–150 km depth could take up to ~15 Ma, but generally < 5 Ma. From studies of short-lived isotopes in arc magmas, the transit time for the return trip made by melts from ~100–150 km depth to the surface has been determined to occur on the order of ~50,000 yr or faster^38,39^, although the recycling of sedimentary components may take longer (~2–4 Ma)^40^. Thus, if fluctuations in arc magmatism imposed by convergence rate changes were tied to physical slab-melt material fluxes, we would expect a lag time on the order of ~5 Ma or less. That the lag times observed do not seem to correspond to the timescales of slab-melt material fluxes is not altogether surprising, because horizontal convergence rate changes do not necessarily correspond to changes in vertical slab sinking rates.

Changes in the horizontal convergence rate will impose changes to the boundary conditions of the mantle wedge, so it is likely that the observed lag times relate to some integrative delay bound to the timescale of mantle wedge convection. We speculate that this integrative delay may comprise the time required to: 1) bring more fertile mantle into the wedge (or conversely to deplete it), 2) for more (or less) vigorous mantle wedge flow to affect the slab temperature (and thus dehydration), and 3) for modifications in mantle wedge convection to exert thermal-viscous changes to both the wedge itself and the base of the overriding lithosphere, enhancing or suppressing decompression melting^35,36^. Although rapid source-to-surface melt transit times suggest that melts may travel via channels^38^, they do not necessarily indicate that these channels develop rapidly, as new melts may utilize existing pathways. Perhaps an additional delay could thus express the time required to open new interconnected networks to accommodate an enhanced melt flux. Responses to convergence rate variations in systems beyond the mantle wedge, for example in the organization of mantle flow around the subducting slab, or in the stress regime of the overriding plate, may be responsible for yet further delays tied to their own timescales.

## Conclusions

Within the Phanerozoic, continental-scale perspective of our experiments, our results suggest that zircon age distributions manifest true fluctuations in arc magmatism, modulated by rates of subduction. We ascribe the rather unexpected ~10–30 Ma lag time between subduction changes and zircon production to processes associated with convection of the mantle wedge, but more work is needed to refine or refute these preliminary conjectures. It remains to be seen how applicable our findings are to more complicated tectonic regions. For example, applied to North America, this analysis yields rather more ambiguous results (Figs S6–S8), likely due, at least in part, to unresolved complexities in the tectonic history of the north-east Pacific^28,30^. Whether fluctuating production predominates over selective preservation on the global scale and in deeper geologic time also remains a critical question. That the global detrital zircon age distribution resembles the global subduction flux^20^ (Fig. 2) with a similar lag time (~15 Ma) to that observed here hints that fluctuating production may also dominate on the global scale, begetting further important questions on the historical cadence of crustal growth and mantle outgassing.

## Methods

### Zircon age frequency distributions

The Phanerozoic zircon age records from South America, Oceania and North America were extracted from the database of Puetz *et al*.^3^, following three filtering steps. 1: All results with ‘Model 1’ (as labeled in the database) ages > 550 Ma were removed. 2: All results with a Model 1 ‘adjusted error ratio’ (as labeled in the database) > 2.7 were removed. 3: All results with a Model 1 ‘adjusted discord ratio’ (as labeled in the database) > 4.0 were removed. A description of these parameters is presented in Puetz *et al*.^3^, and our specific selection of associated cutoff values (in steps 2 and 3) follows the preferred filtering routine of those authors. Following application of these filters, zircon ages (‘Model 1’ ages) were extracted and sorted according to two additional database fields, ‘Continent’ [S_America; Oceania; N_America] and ‘Primary Rock Type’ [igneous; sedimentary; metamorphic]. From these data, the histograms in panels d-g of Figs 3, 4 and S6 were constructed by binning the ages in 5 Ma windows, between 0 and 550 Ma. Accompanying each histogram, Kernel Density Estimates (KDE) computed with a locally variable bandwith (‘adaptive’) were determined from the same age data by implementing the ssvkernel method of Shimazaki and Shinomoto^41^ with a Gaussian window function. The KDEs were then resampled at 5 Ma intervals for comparison with the subduction flux/length data.

### Subduction flux and subduction length

Both time-dependent subduction parameters (flux and length) were calculated from the 410–0 Ma full-plate model of Matthews *et al*.^24^ (M16), which employs continuously closing plate polygons^42^. This plate model is based on the Paleozoic model of Domeier and Torsvik^21^, which explicitly includes the Paleozoic accretion of minor terranes and island arcs to the margins of western South America and eastern Australia. In brief, the Laurentian-derived terrane of Chilenia accreted to the margin of South America at ~390 Ma, followed by the accretion of South Patagonia at ~310 Ma. Notably, with the arrival of Chilenia at ~390 Ma, Laurentia and west Gondwana may have been in close proximity to one another, but neither experienced a major, margin-wide collision then. In eastern Australia, the Gamilaroi-Calliope arc accreted at ~380 Ma, followed by the accretion of the small Gympie-Brook Street terrane (not modelled) at ~250 Ma.

The spatiotemporal distribution of subduction zones was first determined globally for the entire model interval (410–0 Ma) by seeding the dynamic (time-dependent) plate polygon boundaries with a high density of nodes (sub-degree spacing), at which relative plate motions were calculated and averaged over a 1 Ma window, in 10 Ma intervals. 10 Ma intervals were chosen because the Paleozoic full-plate model of Domeier and Torsvik^21^, which underpins the Paleozoic portion of M16, was framed on paleomagnetic data assembled in 10 Ma moving windows. Due to the dynamic nature of the full-plate models and the inclusion of additional data (other than paleomagnetic data) in their construction, a finer temporal resolution is generally still meaningful, but a time-stepping of 10 Ma was selected as a conservative measure. For each time step, the 1 Ma averaged relative motion at each boundary node was decomposed and, for those nodes with a component of convergence, the convergence rate (km/Ma), the associated boundary length segment (km), and the local (node-specific) subducted area flux (km^2^/Ma) were tabulated and collected. The global summation of these local parameters at each time step thus provides an estimate of the time-dependent global length of subduction zones and subducted area flux. However, because we are here only interested in regional subduction (i.e. along western South America or eastern Australia), these global grids are locally resampled at each time-step, wherein only nodes that reconstruct within the specified (and progressively reconstructed) search radius are collected. This was achieved by measuring the great circle distance of each convergent node to the reconstructed center of the search radius, and discarding all nodes with a distance greater than the radius specified.

This approach to calculating subducted area fluxes can be susceptible to over-estimation from non-idealized tectonic processes and model artefacts (e.g. transpressive boundaries, or apparent convergence along a rather long but imperfectly digitized transform boundary). Therefore, two additional filtering steps were applied in the calculation of subduction lengths and fluxes: (1) nodes with a net convergence rate below 0.2°/Ma were excluded, and (2) nodes with a convergent motion component comprising less than 20% of the total motion were excluded. Sensitivity tests conducted by Hounslow *et al*.^20^ reveal that the choice of different cutoff values for these filtering steps affects the magnitude of the computed subduction length/flux, but that their time-dependent trends (which are the focus of the cross-correlations here) remain largely stable (see their Figs S1 and S2); preliminary experiments with such alternative filtering setups yielded results indistinguishable with those presented herein.

### Cross-correlation analysis

Cross-correlation of the subduction parameters (flux, length) against the zircon age distributions was performed as a normalized cross-correlation, effectively a sliding determination of Pearson’s correlation coefficient:

$$\rho =\frac{1}{n}\sum _{{t}_{1}}^{{t}_{n}}\,\frac{1}{{\sigma }_{s}{\sigma }_{z}}(s(t)-\bar{s})(z(t+l)-\bar{z})$$

where *s*(*t*) denotes the subduction parameter (flux or length) as a function of time *t*, *z*(*t* + *l*) is the zircon age frequency at time *t* plus lag time *l*, and *σ*_*s*_, $\bar{s}$ denote the standard deviation and mean of *s*, respectively. In this formulation, a negative lag time *l*, will associate a zircon age frequency observation to an older (preceeding) subduction parameter observation, as noted in the main text. For each lag time *l*, the significance of *ρ* (Pearson’s correlation coefficient) can be determined with reference to standard test values derived from Student’s *t*-distribution, from which the 95% confidence bounds in Figs 3, 4 and S6 were estimated. Lag times were applied to the zircon age frequency distribution (relative to the stationary subduction flux) in 5 Ma steps, corresponding to the width of the zircon age bins. However, the subduction flux was determined at a resolution of 10 Ma, so these time-series comparisons have an inherent error of ±7.5 Ma, and correlations reported at even and odd numbered lag steps can be considered as a semi-independent time-series. These correlation calculations were implemented in Python but were corroborated against standard cross-correlation routines in R. The stronger association between the zircon age distributions and the subduction flux (relative to the association with subduction length) in both Oceania and South America was further substantiated by a relative importance analysis using multiple linear regression, according to the method of Grömping^43^ implemented in R (Fig. S9).

## Electronic supplementary material

Supplementary Materials
