# Supplementary material for: Episodic zircon age spectra mimic fluctuations in subduction
Source: Sci Rep. 2018 Nov 30;8:17471. doi: 10.1038/s41598-018-35040-z (PMC6269492; doi:10.1038/s41598-018-35040-z)
Supplement: Supplementary file 1 — Supplementary Materials [file 41598_2018_35040_MOESM1_ESM.pdf]

Supplementary Materials

Episodic zircon age spectra mimic fluctuations in subduction

Mathew Domeier[1*], Valentina Magni[1], Mark W. Hounslow[2], Trond H. Torsvik[1, 3, 4]

[1]Centre for Earth Evolution and Dynamics (CEED), University of Oslo, Oslo, Norway
[2]Lancaster Environment Centre, Lancaster University, Lancaster, United Kingdom
[3]Geodynamics Team, Geological Survey of Norway, Trondheim, Norway
[4]School of Geosciences, University of Witwatersrand, Johannesburg, South Africa

*Correspondence to mathew.domeier@geo.uio.no

Scientific Reports

Contents: Figures S1-S9, Table S1

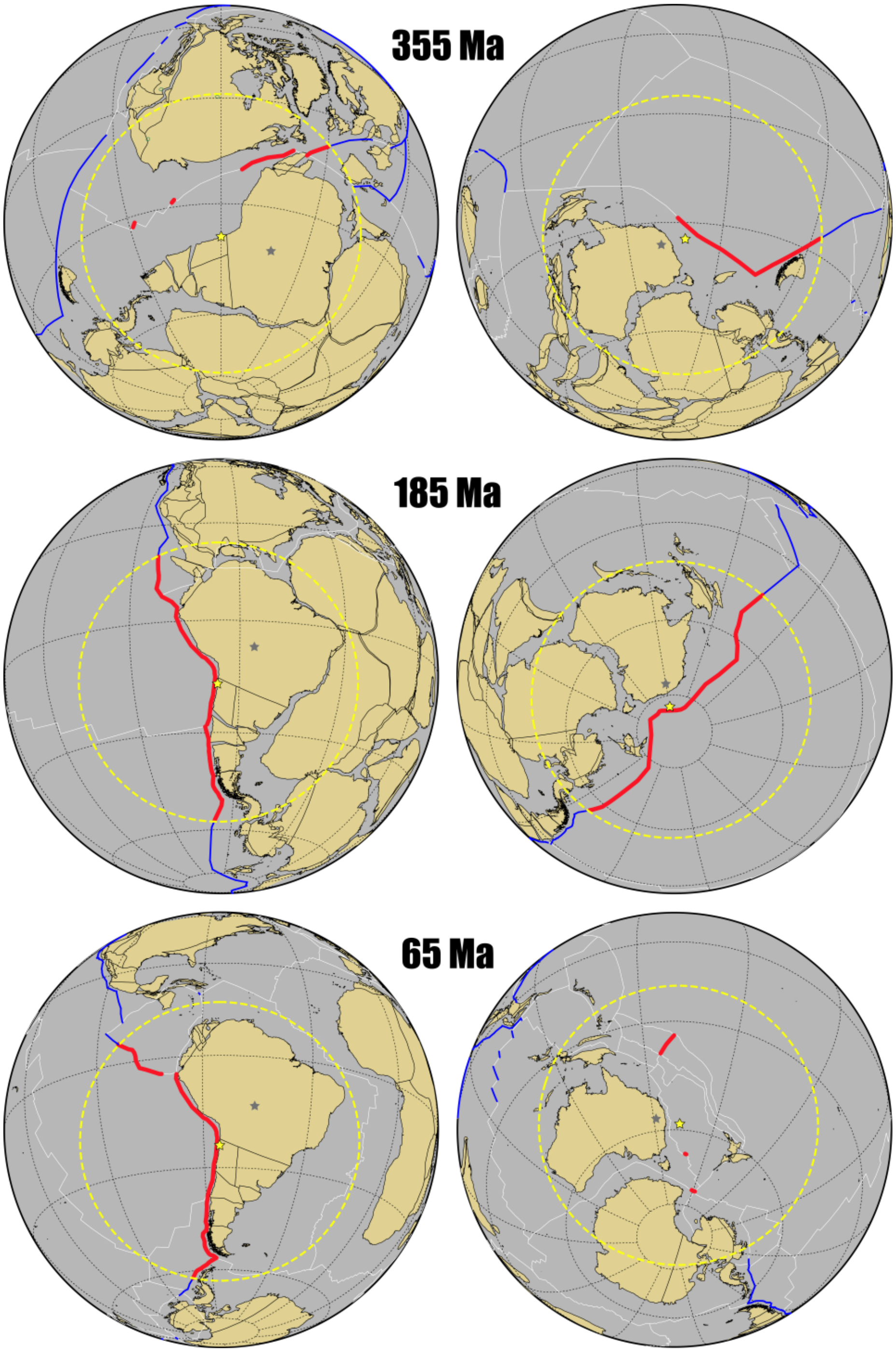

Fig. S1: Example of reconstructions (at 355, 185 and 65 Ma) with subduction segments selected by the initial search setup (weight-based zircon locality centroid and 40° search radius) reconstructed with the continent. Red (blue) subduction zones lie inside (outside) the search radius (yellow dashed line) and would (not) contribute to the calculated subduction flux. Other plate boundaries (divergent, transform) are shown by white lines.

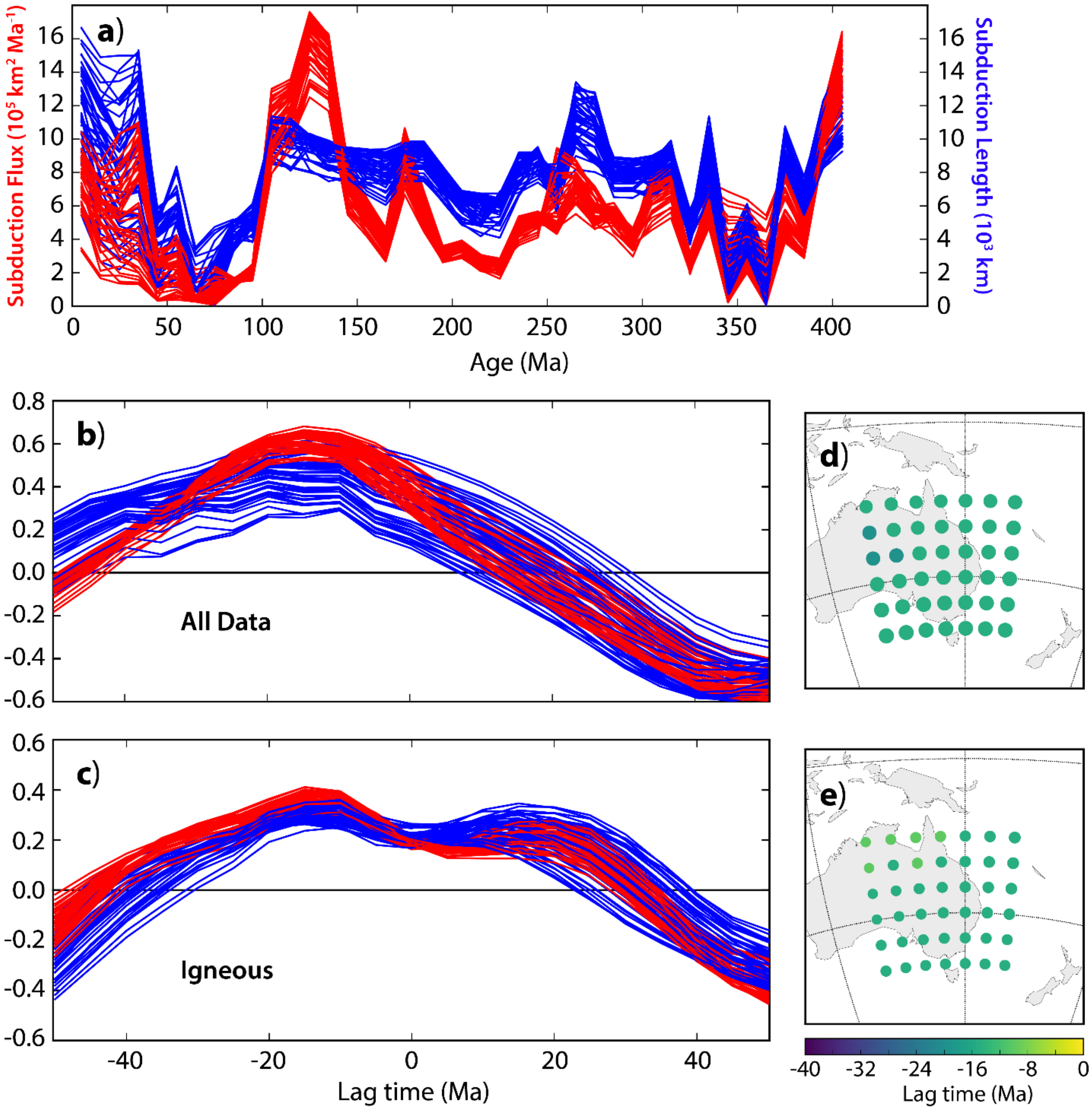

Fig. S2: *Effects of varying the location of the search center for results from Australia.* a) Subduction flux (red) and subduction lengths (blue) calculated with use of different search center locations (shown in panels d, e). b) Resulting cross-correlations between the subduction flux (red) or lengths (blue) and the 'all zircons' age distribution (these show the same thing as the cross-correlation panels in Figs. 3 and 4 of the main text but here the individual bars are connected to show multiple results in one panel). d) The alternative search centers used, with the color showing the lag-time of the maximum correlation and the size of the symbol denoting the magnitude of the correlation. c) and e) same as b) and d) except using the igneous-only zircon data.

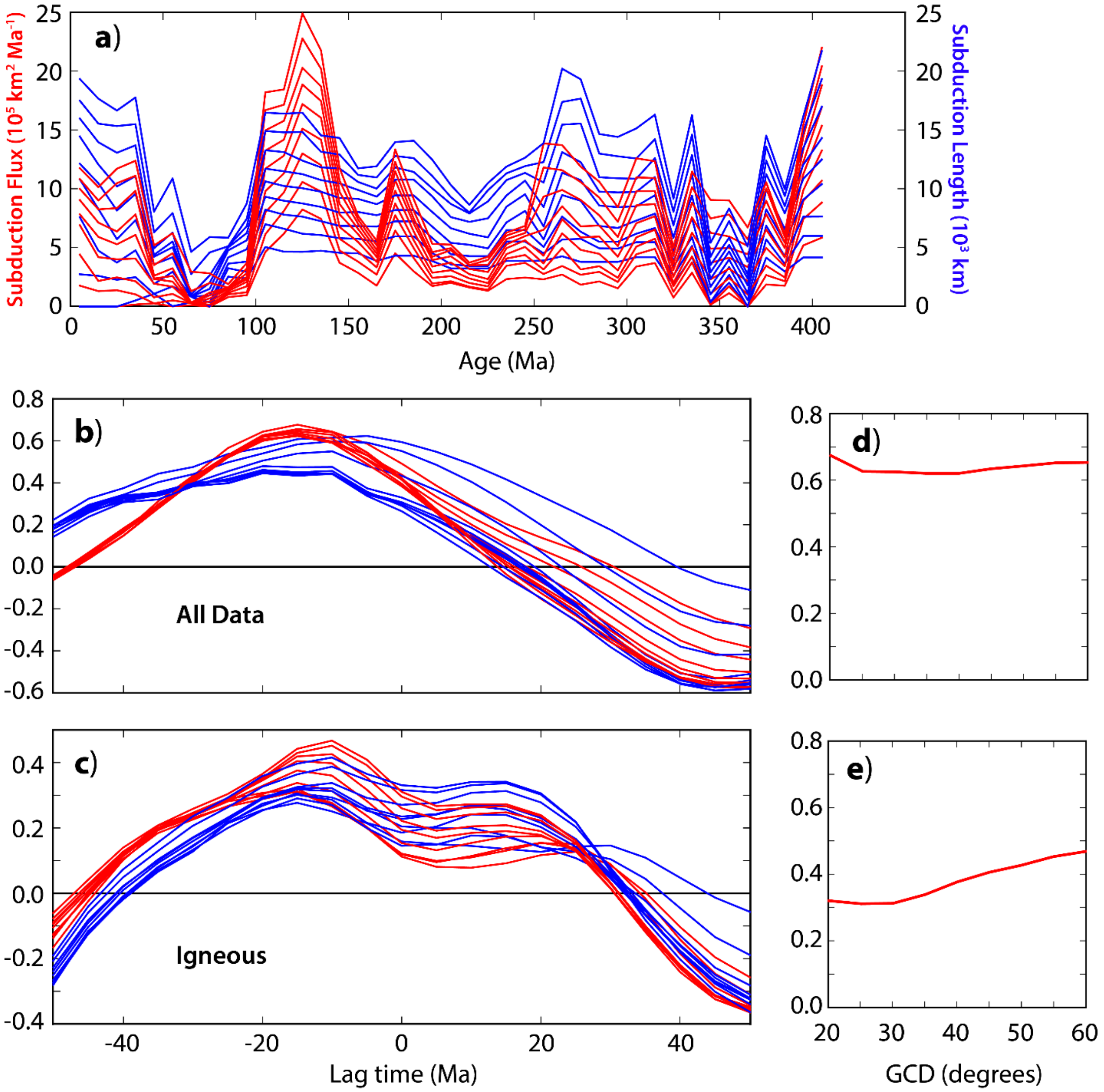

Fig. S3: *Effects of varying the size of the search radius for results from Australia.* a) Subduction flux (red) and subduction lengths (blue) calculated with use of different search radius sizes (shown in panels d, e). b) Resulting cross-correlations between the subduction flux (red) or lengths (blue) and the 'all zircons' age distribution (these show the same thing as the cross-correlation panels in Figs. 3 and 4 of the main text but here the individual bars are connected to show multiple results in one panel). d) The alternative radius sizes tried (5 degree intervals between 20 and 60 degrees), plotted against the maximum correlation achieved. c) and e) same as b) and d) except using the igneous-only zircon data.

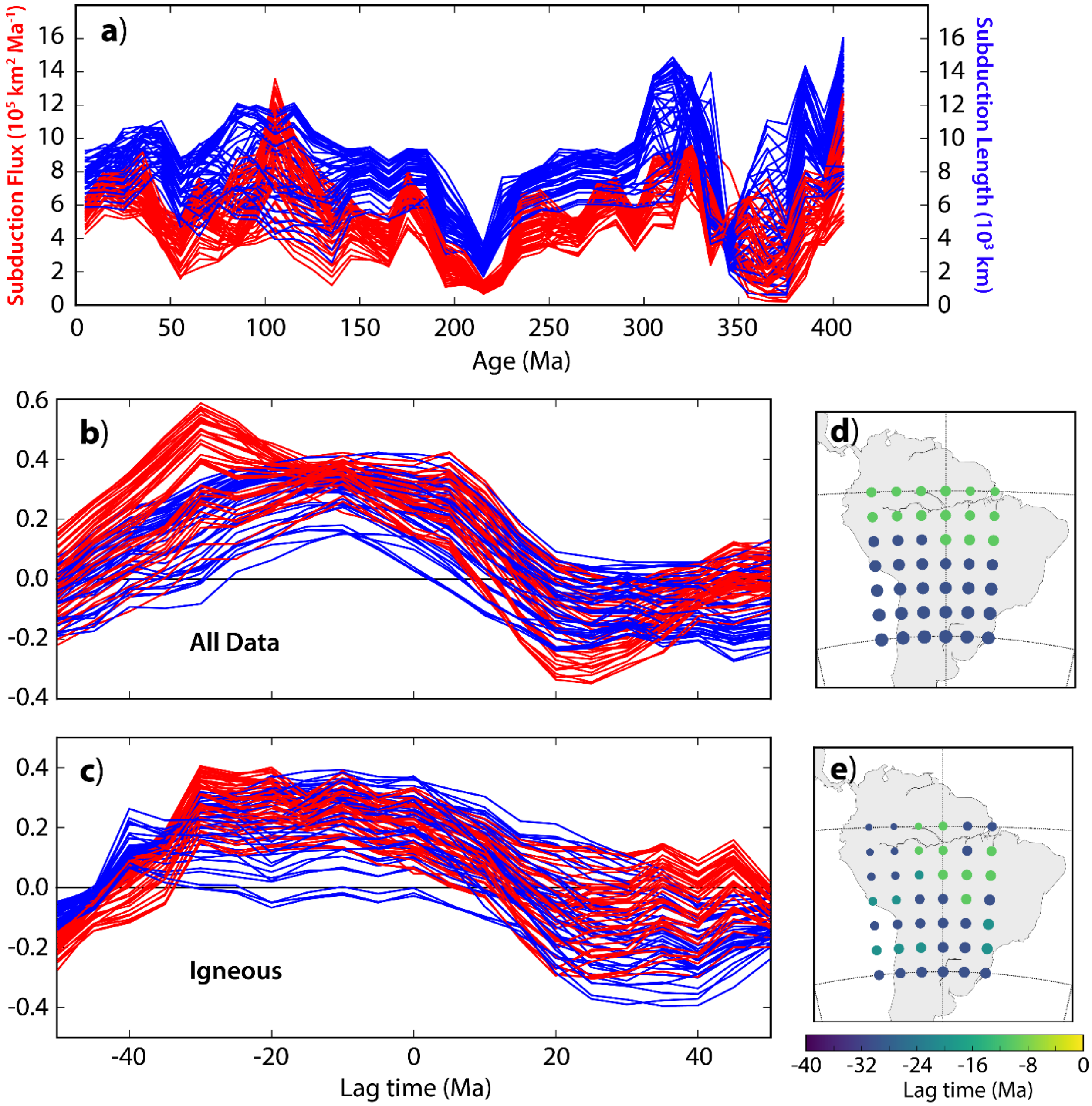

Fig. S4: *Effects of varying the location of the search center for results from South America.* See Fig. S2 for panel descriptions.

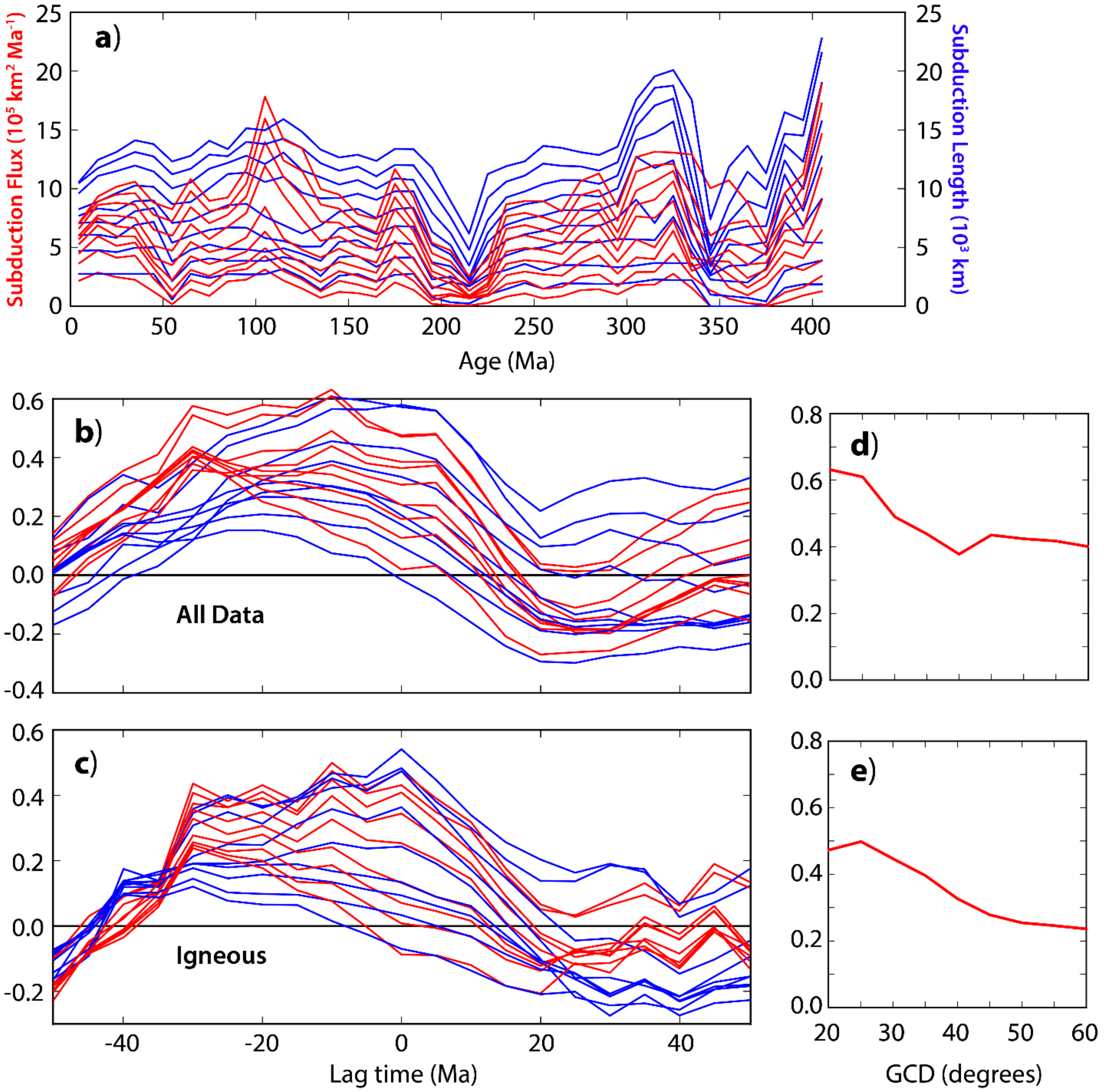

Fig. S5: *Effects of varying the size of the search radius for results from South America.* See Fig. S3 for panel descriptions.

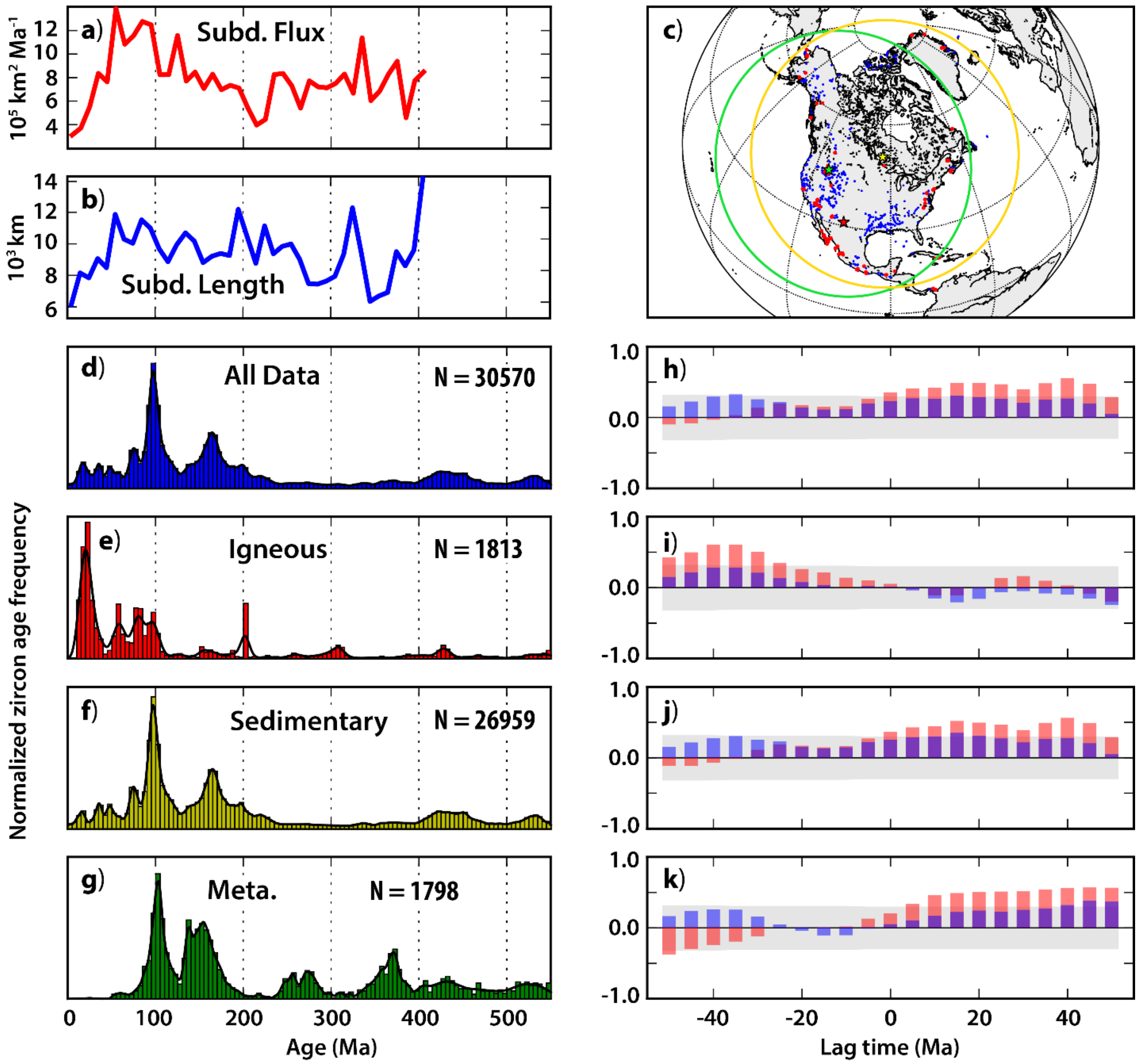

Fig. S6: *Subduction parameters and zircon age distributions from North and Central America.* See Fig. 3 for panel descriptions.

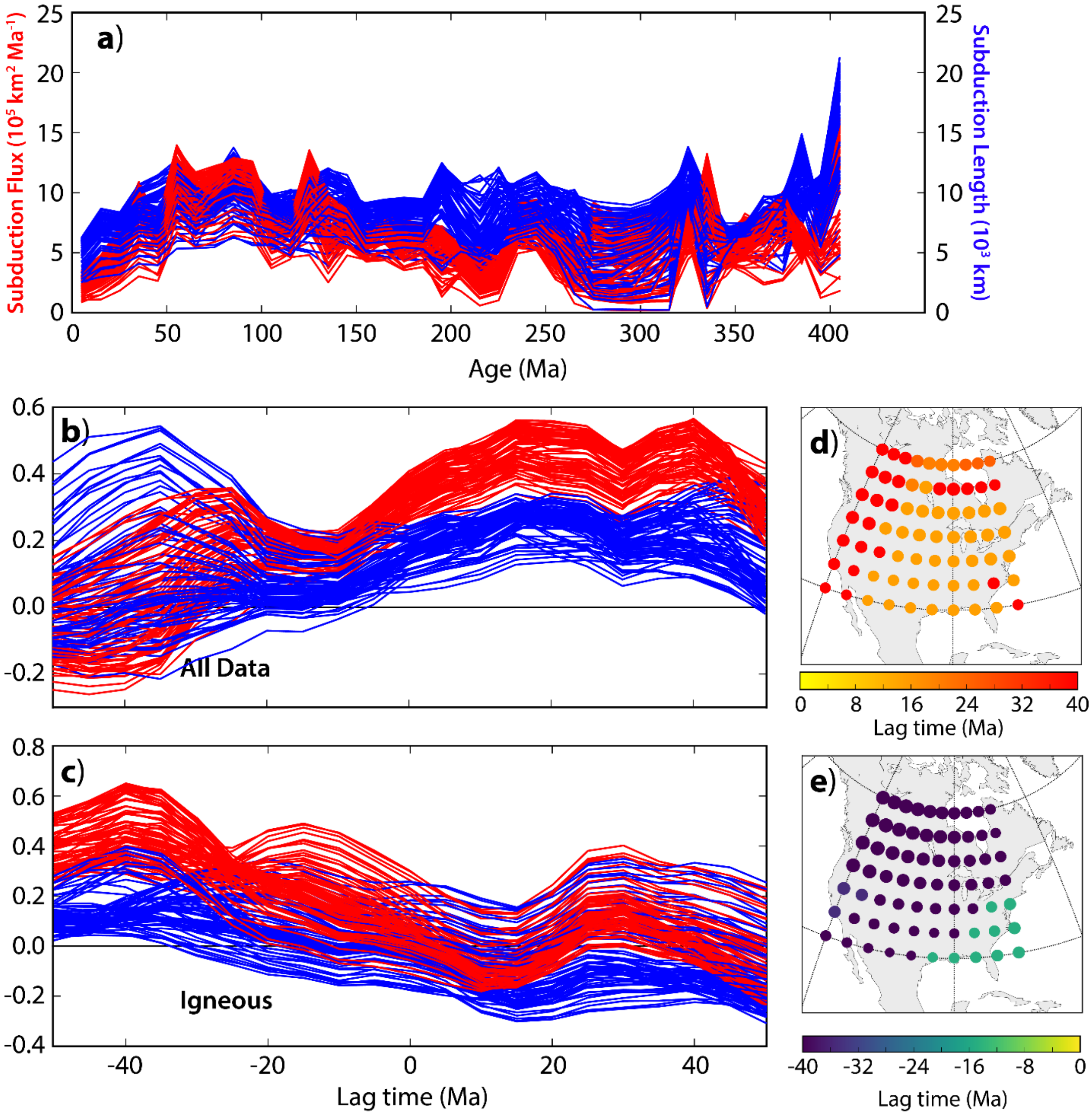

Fig. S7: *Effects of varying the location of the search center for results from North and Central America.* See Fig. S2 for panel descriptions.

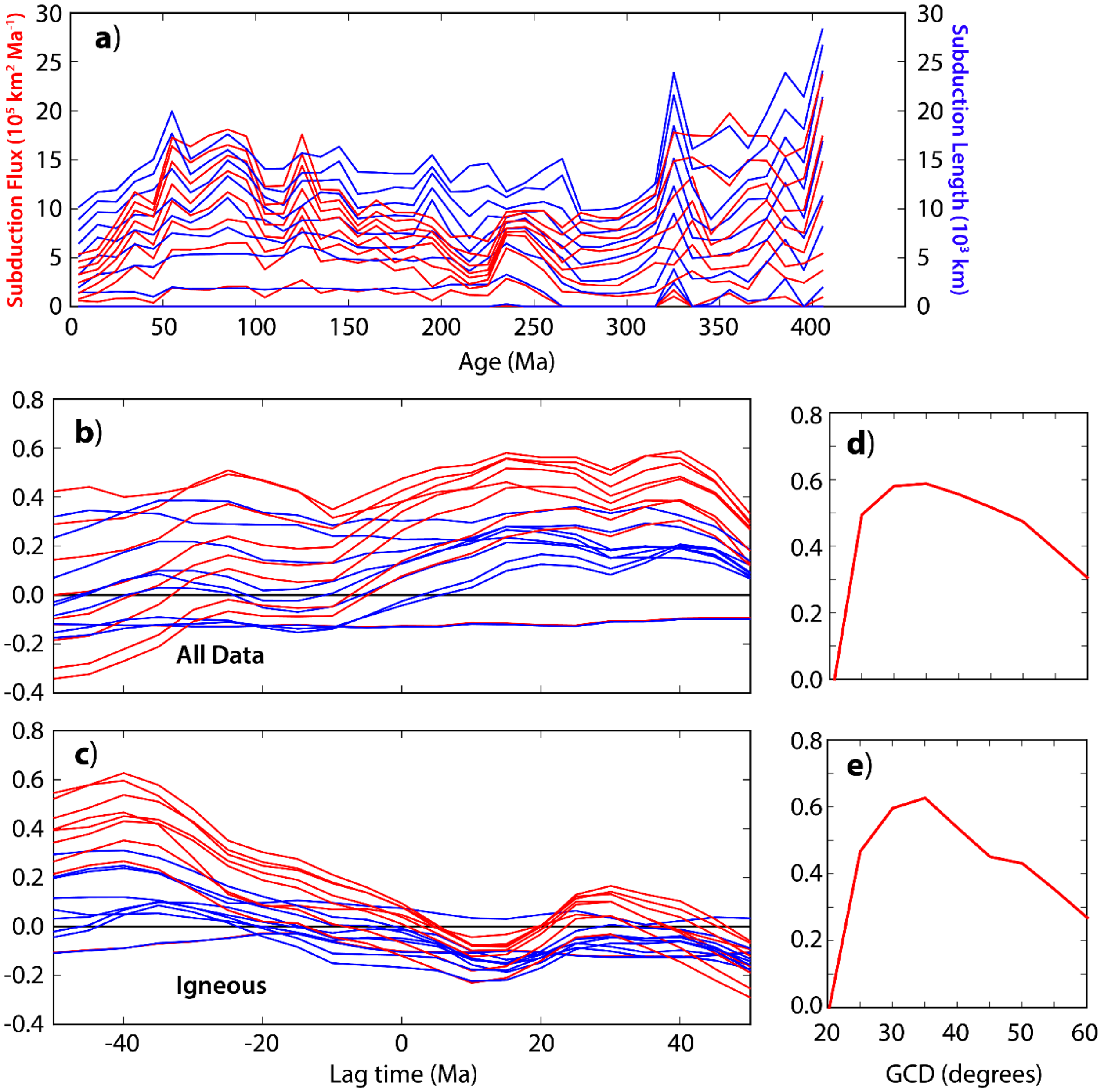

Fig. S8: *Effects of varying the size of the search radius for results from North and Central America.* See Fig. S3 for panel descriptions.

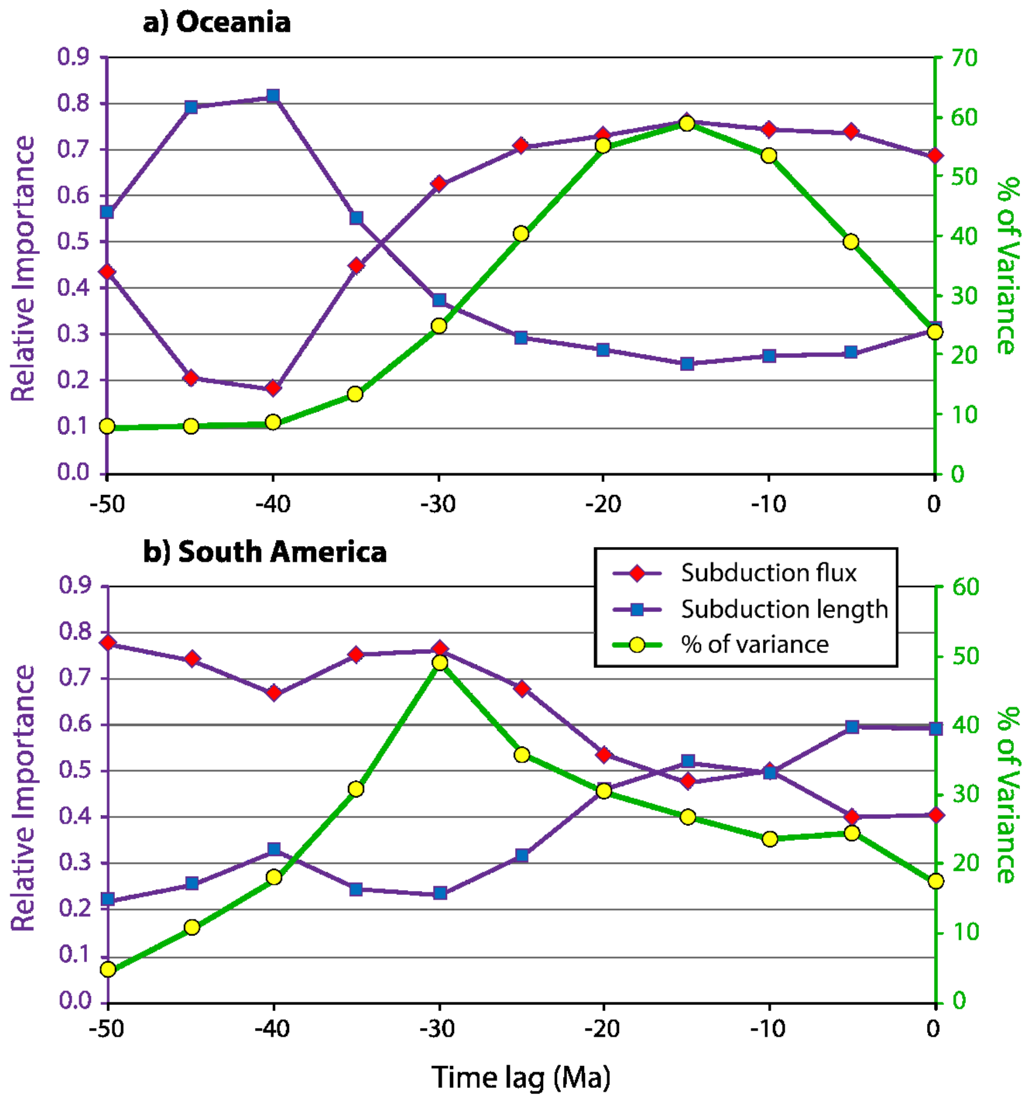

Fig. S9: *Relative importance and percentage variance explained by a multiple linear regression against all zircon data, using subduction flux and length as independent variables.* The subduction flux dominates the contributions when the variance explained is high. Lag time as in cross-correlation Figs. 3, 4 and S6. Subduction parameters > 390 Ma were excluded according to their outlying behavior (Fig. 5). Calculations following the method of Grömping[45], as implemented in R.

Table S1: *Parameters of correlation for linear regression models presented in figure 5.*

South America; -30 Ma lag

Age Range	*Linear Model*	*R^2*
0-180 Ma	y=0.9908x-0.1272	0.551
180-390 Ma	y=0.4238x+0.0778	0.362
post-130 Ma	y=0.8802x-0.0193	0.487

Oceania; -15 Ma lag

Age Range	*Linear Model*	*R^2*
0-180 Ma	y=0.9168x-0.065	0.839
180-390 Ma	y=0.6367x+0.19	0.187
post-130 Ma	y=1.006x-0.0946	0.880
